# Supplementary material for: Vitamin D Modified DSS-Induced Colitis in Mice via STING Signaling Pathway
Source: Biology (Basel). 2025 Jun 18;14(6):715. doi: 10.3390/biology14060715 (PMC12190092; doi:10.3390/biology14060715)
Supplement: Supplementary file 1 [file biology-14-00715-s001.zip › Supplementary Materials S1: 16s-report of gut microbiota/Groups/diff_analysis/Correlation/Genus-GMM.pdf]

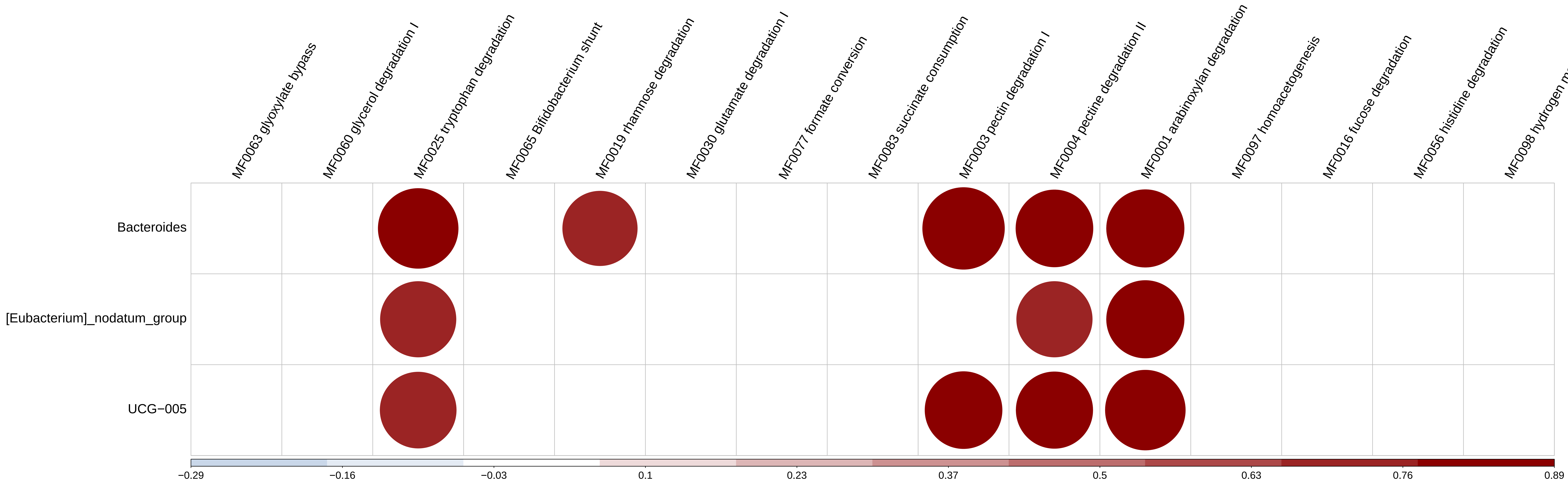

MF0063 glyoxylate bypass
MF0060 glycerol degradation I
MF0025 tryptophan degradation
MF0065 Bifidobacterium shunt
MF0019 rhamnose degradation
MF0030 glutamate degradation I
MF0077 formate conversion
MF0083 succinate consumption
MF0003 pectin degradation I
MF0004 pectine degradation II
MF0001 arabinoxylan degradation
MF0097 homoacetogenesis
MF0016 fucose degradation
MF0056 histidine degradation
Bacteroides
[Eubacterium]_nodatum_group
UCG−005
−0.29
−0.16
−0.03
0.1
0.23
0.37
0.5
0.63
0.76
0.89
